# Adaptive primal–dual Q-learning for electric vehicle route optimization on real-world charging networks

**DOI:** 10.1038/s41598-026-49124-8

**Published:** 2026-07-23

**Authors:** Sarvesh Ram Kumar, Rayappa David Amar Raj, Archana Pallakonda, K. Sreelakshmi, K. Krishna Prakasha

**Affiliations:** 1 Amrita School of Artificial Intelligence, Amrita Vishwa Vidyapeetham, Coimbatore, India; 2 Department of Computer Science and Engineering, National Institute of Technology Warangal, Hanamkonda, Telangana 506004 India; 3 Manipal Institute of Technology, Manipal Academy of Higher Education, Manipal, 576104 India

**Keywords:** Energy science and technology, Engineering, Mathematics and computing

## Abstract

Electric Vehicles (EVs) are emerging as sustainable alternatives to internal combustion engine vehicles; however, efficient route planning remains a major challenge due to limited driving range, sparse charging infrastructure, and variable energy consumption patterns. Traditional shortest-path algorithms, such as Dijkstra’s and A*, often fail to account for EV-specific factors, including charging station availability, connector compatibility, and energy constraints. This study presents a comprehensive EV route optimization framework that integrates reinforcement learning (RL) with graph-based methods. A novel Dual Q–Adaptive Weighting model that balances reward and cost through a primal–dual learning mechanism is proposed. The framework learns energy-aware routing strategies from historical navigation experience. The model is compared against standard RL approaches—Q-Learning and Double Q-Learning—as well as enhanced variants of A* and Dijkstra’s algorithms that incorporate charging density and time-penalty considerations. Real-world EV charging infrastructure data from the Alternative Fuels Data Center (AFDC) and Placekey datasets are used to construct a clustered navigation graph via DBSCAN. Experimental results across multiple intercity routes show that the proposed Dual Q–Adaptive model achieves the highest route accuracy of 78.66%, outperforming Double Q-Learning (76.27%), Q-Learning (77.52%), and traditional A* (74.26%) and Dijkstra (60.92%) algorithms. A* and Dijkstra with modifications, use fewer charging stops than traditional algorithms. The Improvised algorithms provide substantial improvements over their baseline counterparts. The results demonstrate that reinforcement learning integrated with graph-theoretic optimization can enable scalable, infrastructure-aware, and efficient EV route planning.

## Introduction

Electric vehicles (EVs) are rapidly gaining popularity worldwide for their environmental and economic benefits^1^. However, challenges such as limited battery capacity, variable energy consumption, and sparse charging infrastructure pose significant obstacles for efficient route planning. Traditional shortest-path algorithms like Dijkstra and A* often fall short in EV-specific scenarios, as they do not consider dynamic factors like charging station availability, energy consumption, or connector type. Inadequate route planning not only leads to range anxiety among EV drivers but also affects the overall efficiency and reliability of EV travel. Unlike conventional vehicles, EVs must carefully balance travel distance, energy consumption, and charging opportunities to complete long trips successfully. Moreover, variations in charger availability, charging speeds, and connector compatibility further complicate routing decisions. As EV adoption continues to grow, robust and intelligent route planning systems become essential. These systems help optimize battery usage, reduce waiting times at charging stations, and improve the overall user experience^2^. Effective EV route planning directly supports the wider adoption of sustainable transportation by enhancing both convenience and operational efficiency.

Early research on EV route planning focused primarily on minimizing travel time using simplified models. For instance, a fast clustering and pruning method for reducing road network size is proposed in^3^, enabling real-time use of exact algorithms like Dijkstra without significant accuracy loss. However, it ignores key EV constraints such as state-of-charge (SoC) and battery usage, reducing practical applicability. A computationally efficient energy consumption model using high-level vehicle specifications, validated against FASTSim and ideal for real-time applications, was introduced in^4^. However, its energy estimates are not integrated into routing decisions. This limits its usefulness for practical route planning. To better match real-world EV behavior, other studies incorporated topography and battery usage into routing decisions. An energy-optimal routing system was presented in^5^. It integrates battery degradation and elevation data using a modified Yen’s algorithm. This approach balances travel time with battery wear, improving long-term performance, but lacks logic for dynamic recharging decisions mid-route, making it less adaptable. Real-world simulations have also been employed to understand EV routing challenges. An evaluation of 60 real EV routes in Germany was done in^6^, revealing travel delays due to sparse charging infrastructure. The findings underscore the impact of vehicle efficiency and initial SoC on journey duration. However, no route optimization or decision-support logic was implemented. Likewise, emergency scenarios were modeled in^7^ where EVs deliver electricity to isolated regions, comparing travel distance vs. energy-optimal paths and demonstrating up to 9.4% energy savings. But the model assumes static delivery routes and does not adapt to dynamic conditions.

Several studies extend EV routing by incorporating electricity pricing and operational constraints. A dynamic programming framework with time-of-use pricing was proposed in^8^, increasing fast-charger utilization by 25%, though it ignores real-time battery usage and SoC constraints. Similarly,^9^ integrates crowd-sensed traffic data and queue theory to optimize routes and charging schedules, but does not fully consider battery levels or charger placement. Metaheuristic approaches have also been widely used. Adaptive particle swarm optimization with nonlinear energy models was introduced in^10^, while an improved ant colony optimization method in^11^ minimizes travel time and charging costs. However, these approaches often require significant parameter tuning and become computationally expensive for large networks. Evolutionary algorithms have further improved EV routing. A hybrid genetic algorithm with dynamic Dijkstra was proposed in^12^ to handle variable speeds and in-transit charging. Another approach, the reinsertion genetic algorithm (RI-GA), achieved up to 12.33% energy savings and faster convergence^13^. Nevertheless, these methods still struggle to scale efficiently for large graphs.

Research has also explored integrated transport–energy systems. A collaborative cyber-physical framework in^14^ enables coordinated multi-EV charging across transport and power networks. The Smart Search of Route and Charging (S2RC) system^15^ incorporates SoC, traffic conditions, charging station availability, and reservation systems, though it lacks adaptive learning capabilities. EV health monitoring and range prediction have also been explored to support route planning^16^. Recent work investigates reinforcement learning for EV routing. Deep RL has been applied to the electric vehicle routing problem with time windows^17^. Another hybrid approach combining GRU-based electricity price forecasting with an A*-Dijkstra-SAA framework achieved up to 18% charging cost reductions^18^. While a two-stage deep RL training strategy improves solution quality in practical scenarios^19^. Recent reports such as the Global EV Outlook^20^ highlight the rapid growth of EV adoption worldwide. Machine learning methods are also used for charging demand prediction, including physics-informed graph learning models^21^. Public datasets such as UrbanEV^22^ further support data-driven EV research.

While recent contributions have improved EV routing via predictive models and multi-objective optimization, they still lack lightweight, interpretable learning mechanisms that directly adapt to SoC constraints and charger availability through real-time interaction. Our work introduces a primal–dual reinforcement learning framework that outperforms both classical and improvised graph-search baselines. Using Q-Learning, Double Q-Learning, and a novel Dual Q–Adaptive Weighting formulation, the agent dynamically balances travel efficiency and energy constraints without predefined heuristics or metaheuristic tuning. Unlike GRU-based or deep RL architectures that require substantial computational infrastructure, the proposed tabular, primal–dual strategy remains computationally lightweight and fully interpretable, yet delivers superior route optimization performance.

The major contributions of this work are as follows:

- A Dual Q-Adaptive Weighting reinforcement learning framework embedded in an end-to-end EV routing pipeline. The model learns optimal routes through interaction with the environment while considering SoC, energy cost, and charging station availability, outperforming standard algorithms and RL baselines.
- Comprehensive benchmarking across seven models, including two classical baselines (Dijkstra and A*), two energy-aware variants, the proposed Dual Q-Adaptive Weighting RL model, and two standard RL methods (Q-Learning and Double Q-Learning).
- Integration of a large-scale real-world charging station network, including node clustering, connector-type constraints, and SoC-constrained transitions to better reflect realistic EV driving conditions.
- Demonstration that the proposed primal–dual RL approach improves route efficiency, constraint satisfaction, and adaptability in sparse-charging environments compared to classical and standard RL methods.
- Discussion of potential applications in EV navigation systems, smart transportation networks, and energy-aware routing tools, particularly for regions with limited charging infrastructure.

Unlike deep RL approaches that rely on neural approximators and are often less suited and computationally more expensive when applied to large discrete routing graph structures, our method uses a tabular primal–dual formulation that is naturally aligned with the discrete EV-routing state space. This avoids function-approximation errors, improves training stability, and enables full interpretability of the learned policy. The Lagrangian-driven adaptive weight provides explicit control over the trade-off between route efficiency and charging constraints.

## Dataset description

The *Comprehensive Database of Electric Vehicle Charging Stations in the U.S. and Canada* from the Placekey organization^23^ has been used in this work. This dataset, sourced from the U.S. Department of Energy’s Alternative Fuels Data Center, contains detailed information on charging stations across the United States and Canada. It includes station locations, types of available charging connectors, and n tools for mapping routes and locating nearby stations. For the purposes of this study, from Table 1 the Latitude, Longitude, and EV Connector Types fields have been used extensively for training models in EV route planning scenarios. The Graph node structure is built using the latitude and longitude of the charging station.

**Table 1:** Charging Station Dataset Field Descriptions.

Field Name	Description
EV Workplace Charging	Indicates availability of workplace charging for electric vehicles.
ID	Unique identifier for the station.
Access Code	Code indicating the level of access to the charging station.
Latitude	Geographical latitude of the station.
Country	Country where the station is located.
ZIP	ZIP code of the station location.
Longitude	Geographical longitude of the station.
Groups With Access Code	Groups that can access the station based on access code.
EV Pricing	Pricing details for using the charging station.
Status Code	Code representing the operational status of the station.
Station Name	Name of the charging station.
Facility Type	Type of facility where the station is located.
EV Network Web	Website of the electric vehicle network.
EV Connector Types	Types of connectors available at the charging station.
City	City where the station is located.
EV Level2 EVSE Num	Number of Level 2 EV Supply Equipment available.
Maximum Vehicle Class	Maximum class of vehicle that can be accommodated by the station.
EV Network	Name of the network the charging station belongs to.
Geocode Status	Status of the geocoding process for the station’s location.
State	State where the station is located.
Fuel Type Code	Code representing the type of fuel available at the station.

## Proposed methodology

**Fig. 1:**
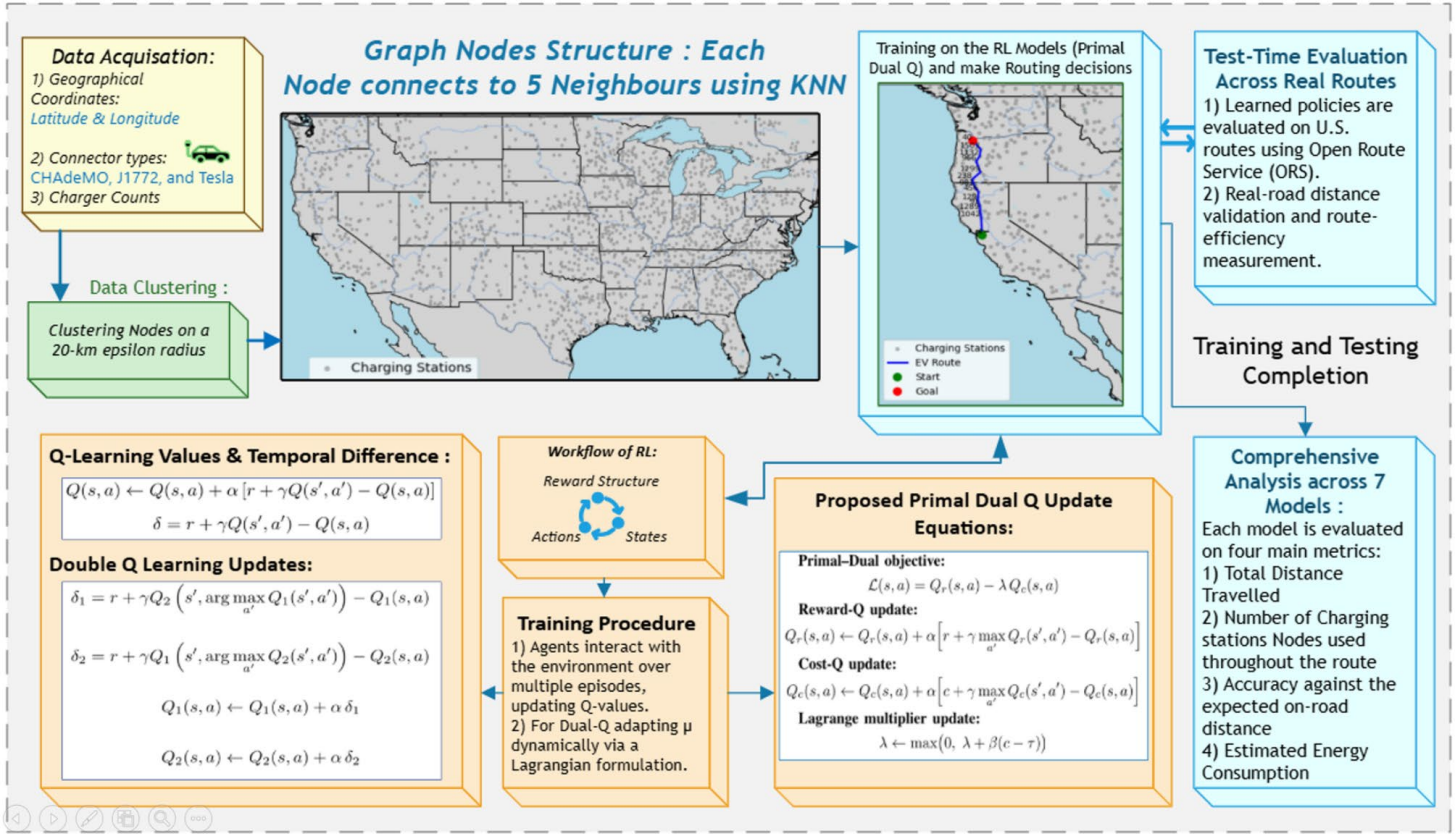
The Learning Process followed by the RL models. Maps generated using Cartopy v0.24.1^25^ with Natural Earth map data^26^.

This section details the implementations of training and testing of Reinforcement Learning models (Primal-Dual Q, Q-Learning and Double Q-Learning), standard and improvised versions of A* and Dijkstra which are used to plan routes for Electric Vehicles. The Overall flow of the work is shown in Figure 1.

### Data acquisition and graph construction

A nation-wide catalogue of public charging stations was obtained from Alternative Fuels Data Center. Each record provides us with the Latitude of the charging stations ($\phi$), Longitude of the charging station ($\lambda$), the available Connector types in the charging station (e.g., J1772, CCS-2, CHAdeMO) and the Number of ports available at the charging station $p$. For graph construction and RL training, the Haversine distance is used to compute edge weights, providing a fast, consistent approximation suitable for large-scale spatial graphs and high-frequency environment interactions during training. For evaluation and final route validation, real-world driving distances are obtained using OpenRouteService (ORS)^24^, ensuring accurate comparison to actual road-network distances. All maps presented in the figures were generated using the Cartopy library^25^ with base map data from Natural Earth^26^. The Graph Construction is done with the help of Latitudes ($\phi$) and Longitudes ($\lambda$) of the Charging stations. To reduce the graph density, stations are clustered with an $\varepsilon$-ball radius of 20 km, i.e., two stations $i$ and $j$ become part of the same node if

1 $$\begin{aligned}&a = \sin ^2\left( \frac{\phi _i - \phi _j}{2} \right) + \cos (\phi _i)\cos (\phi _j)\sin ^2\left( \frac{\lambda _i - \lambda _j}{2} \right) \end{aligned}$$

2 $$\begin{aligned}&d(i,j) = 2 R_\oplus \cdot \arcsin \left( \sqrt{a} \right) \end{aligned}$$

The epsilon parameter for DBSCAN was set to 20 km to balance spatial clustering and network connectivity. Smaller epsilon values may produce fragmented clusters that reduce connectivity between charging stations, while larger values may over-aggregate stations and reduce spatial resolution in the routing environment. The selected value provided a practical compromise that preserved connectivity across the large geographic region while maintaining realistic charging station spacing. The Haversine distance between two stations $i$ and $j$ is computed using Equations (1) and (2), where $R_\oplus$ denotes the Earth’s radius (6371 km), and $\phi$, $\lambda$ are the latitudes and longitudes in radians. Each node $v_i \in V$ represents a charging station with a geographic position $\textbf{x}_i = (\phi _i, \lambda _i)$, where $\phi$ and $\lambda$ denote latitude and longitude, respectively. To construct the graph, we connect each node to its $k$ nearest neighbors using geodesic (Haversine) distance as the metric.The set of $k$-nearest neighbors for node $v_i$ is defined in Equation  (3)

3 $$\begin{aligned} \mathcal {N}_k(v_i)&= \underset{v_j \in V \setminus \{v_i\}}{\operatorname {argmin}^{k}} \; d(v_i, v_j) \end{aligned}$$

To model both spatial proximity and the utility of charging capacity, the edge weight $w_{ij}$ between nodes $v_i$ and $v_j$ is computed in Equation  (4)

4 $$\begin{aligned} w_{ij}&= \frac{d(v_i, v_j)}{1 + c_j} \end{aligned}$$

where $d(v_i, v_j)$ is the geodesic distance between $v_i$ and $v_j$, and $c_j$ is the number of available chargers at node $v_j$.

The resulting edge set $E$ is constructed in Equation  (5)

5 $$\begin{aligned} E&= \left\{ (v_i, v_j, w_{ij}) \;\bigg |\; v_j \in \mathcal {N}_k(v_i) \right\} \end{aligned}$$

This approach creates a charger-aware KNN graph, prioritizing nearby stations with greater charging availability .After clustering and 5-nearest-neighbor construction, the resulting nationwide graph contains approximately 2076 representative charging-station nodes. So with the graph constructed and the proposed RL, classical algorithms learn are trained and tested to find the most optimal path from the start to the destination locations.

### Implementations of reinforcement learning models: primal-dual Q learning, Q-learning & double Q-learning

The implementation of the proposed Primal–Dual Q model requires a well-defined EV routing environment, a reward structure that reflects range, charging, and distance constraints, and sufficient interaction for the agent to learn effective policies. The primal–dual formulation introduces an adaptive Lagrangian weighting that balances reward and cost during training, enabling flexible and interpretable control of routing behavior. For completeness, standard Q-Learning and Double Q-Learning models are also implemented within the same environment, following their respective update mechanisms, and serve as baseline reinforcement learning approaches for comparison.

#### Environment description

We design a custom reinforcement learning (RL) environment for electric vehicle (EV) route planning on a spatial graph. The agent’s goal is to navigate from a given start node to a goal node while optimizing travel efficiency and charging utility. The environment is defined over a graph $G = (V, E)$, where each node $v \in V$ has a geographic position and may include metadata such as charger availability and supported connector types. An episode starts at a designated start node and terminates when the agent reaches the goal node. At each timestep, the agent selects an action corresponding to moving to a neighboring node. The environment returns the new state, a scalar reward, and a boolean indicating whether the goal has been reached.

#### Problem formulation

Formally, the EV routing task is modeled as a constrained Markov decision process (CMDP), where states correspond to graph nodes and actions correspond to transitions to neighboring nodes. The objective is to learn a policy that maximizes cumulative routing reward while limiting cumulative travel cost. This can be expressed as the constrained optimization problem:

6 $$\begin{aligned} \max _{\pi } \; \mathbb {E}_{\pi }\!\left[ \sum _{t=0}^{T}\gamma ^t R(s_t,a_t)\right] \quad \text {s.t.} \quad \mathbb {E}_{\pi }\!\left[ \sum _{t=0}^{T}\gamma ^t C(s_t,a_t)\right] \le \tau \end{aligned}$$

where $R(s_t,a_t)$ denotes the routing reward and $C(s_t,a_t)$ represents the travel cost. The proposed Primal–Dual Q-learning approach approximates this objective using a dual variable $\mu$, which adaptively balances reward and cost during learning.

#### Reward function description

As shown in Table 2, the Reward function is setup such that it would prefer routes with less distance travelled, prefer nodes with necessary connector type of the electric vehicle and avoid detours and delays as much as possible.

**Table 2:** Reward Function Design in the RL Environment.

Component	Description	Reward
Distance Penalty	Penalizes longer travel based on the geodesic distance between current and next node.	$-d(v_i, v_j)$
Connector Reward	Applies a bonus if the destination node has a charger with the agent’s preferred connector type, or a small penalty otherwise.	$+10$ (preferred), $-3$ (non-preferred)
Goal Proximity Reward	Encourages movement toward the goal by rewarding reduction in distance to the goal node.	$\Delta d = d_{\text {prev}} - d_{\text {new}}$
Delay Penalty	Discourages excessive steps through an exponential time penalty, increasing as the episode progresses.	−0.1 × 1.05^steps^ (capped at − 900)
Loop Penalty	Imposes a penalty each time a node is revisited, increasing with visit count.	$-100 \times \text {visits}$
Goal Reward	Grants a large reward for reaching the goal, signifying task completion.	$+10000$

#### Learning algorithm of primal-dual Q-learning

Reinforcement Learning models work upon their experience and hence need training for exploration and exploit the best actions to get maximum rewards. As shown in **Algorithm 1**, the proposed *Primal–Dual Q-Learning with Adaptive Weighting* model maintains two distinct value functions: a reward-based Q-table $Q_r(s,a)$ and a cost-based Q-table $Q_c(s,a)$. The agent selects actions using a Lagrangian-combined score that balances efficiency against constraint violations. The balance is controlled by a dual variable $\mu$, which is updated through gradient-based dual ascent. For all reinforcement learning experiments, a learning rate of $\alpha = 0.1$, a discount factor of $\gamma = 0.9$, and an $\epsilon$-greedy exploration strategy with $\epsilon$ value being 0.2 is used.

During each transition from state $s$ to $s'$ via action $a$, the reward and cost components are updated using separate temporal-difference (TD) targets. The combined Lagrangian score used for action selection is given in Equation (7). The primal updates for reward and cost values follow Equations (8) and (9), while the dual update rule for $\mu$, enforcing the cost constraint, is given in Equation (10).

7 $$\begin{aligned} & \mathcal {L}(s,a) = Q_r(s,a) - \mu \, Q_c(s,a) \end{aligned}$$

8 $$\begin{aligned} & Q_r(s,a) \leftarrow Q_r(s,a) + \alpha \Big [ r + \gamma \max _{a'} Q_r(s',a') - Q_r(s,a) \Big ] \end{aligned}$$

9 $$\begin{aligned} & Q_c(s,a) \leftarrow Q_c(s,a) + \alpha \Big [ c + \gamma \max _{a'} Q_c(s',a') - Q_c(s,a) \Big ] \end{aligned}$$

The dual variable $\mu$ adjusts according to the magnitude of constraint violation $(c - \tau )$, where $\tau$ is the target cost threshold:

10 $$\begin{aligned} \mu \leftarrow \max \Big ( 0,\, \mu + \eta (c - \tau ) \Big ) \end{aligned}$$

This update corresponds to a dual-ascent step commonly used in primal–dual optimization methods, where the dual variable increases when the cost constraint is violated and decreases when the constraint is satisfied. This primal–dual structure allows the agent to dynamically learn trade-offs between route efficiency and constraint satisfaction (e.g., SoC safety margin, energy cost, or charging availability), producing interpretable and constraint-aware routing policies.

**Algorithm 1:**
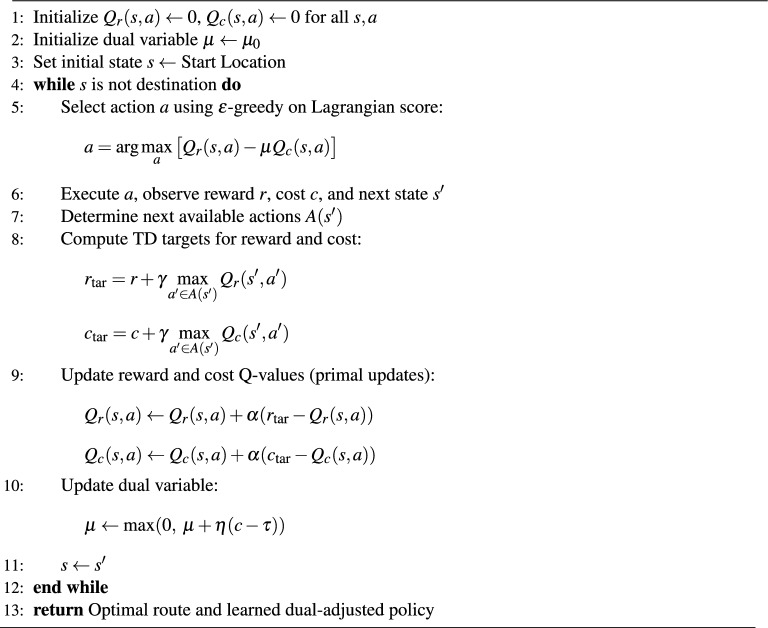
Primal-Dual Q Learning with Adaptive Weighting RL Algorithm.

#### Learning algorithm of Q and double Q learning

As shown in **Algorithm** 2, the Learning Algorithm of Q-Learning model only uses a single agent for selecting and evaluating the actions. During the training of the Q-Learning Model, the Q-value is updated using the temporal difference(TD) learning rule. Specifically, when the agent transitions from state $s$ to a new state $s'$ by taking action $a$ and receiving reward $r$, and then selects a next action $a'$, the Q-value is updated using the Equation (11). The Temporal difference is calculated using Equation (12).

11 $$\begin{aligned} & Q(s, a) \leftarrow Q(s, a) + \alpha \left[ r + \gamma Q(s', a') - Q(s, a) \right] \end{aligned}$$

12 $$\begin{aligned} & \delta = r + \gamma Q(s', a') - Q(s, a) \end{aligned}$$

**Fig. 2:**
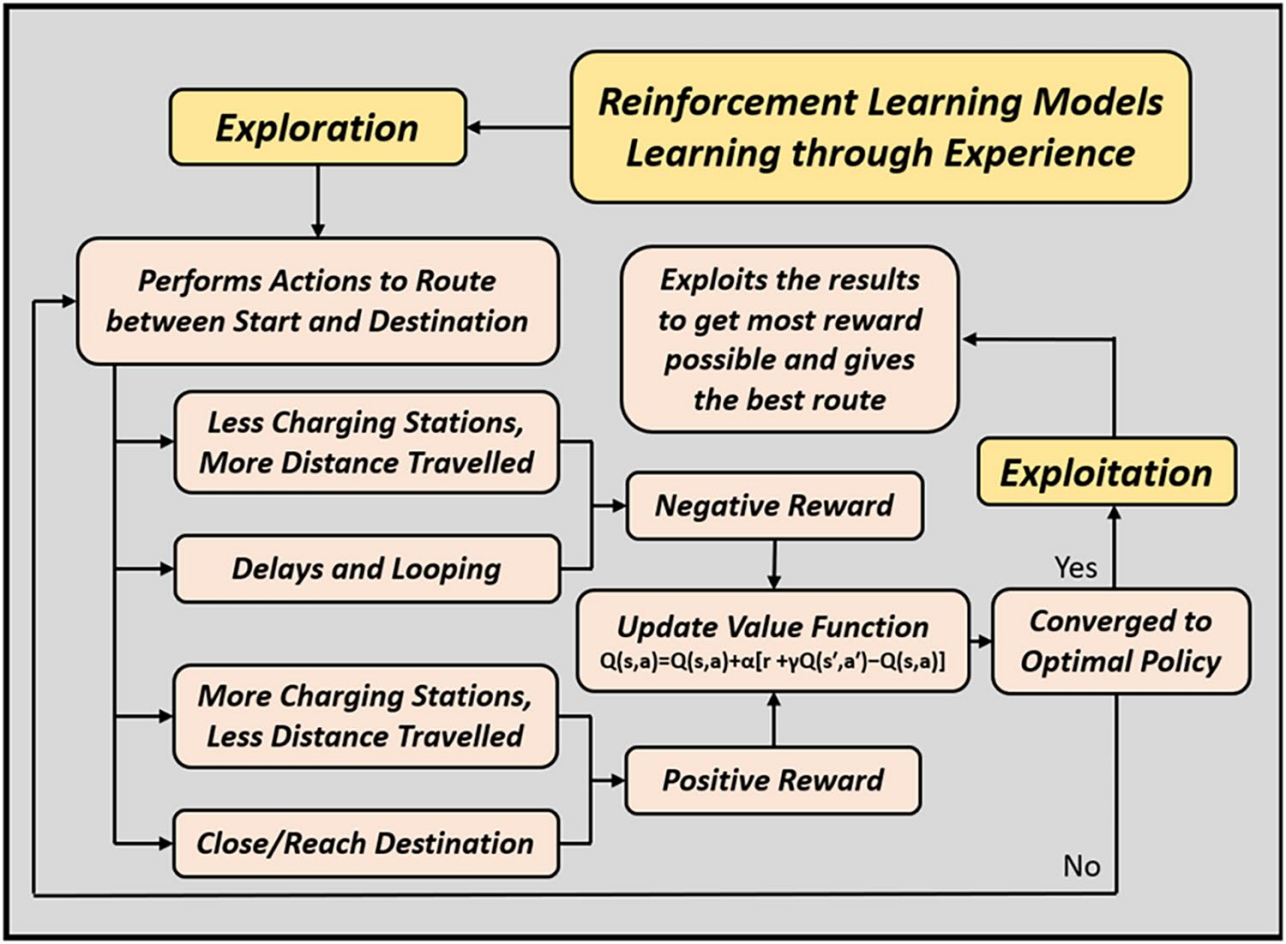
The Learning Process followed by the RL models.

In Equation (12), $\alpha$ is the learning rate and $\gamma$ is the discount factor. The use of $Q(s', a')$ rather than $\max _{a'} Q(s', a')$ indicates that this implementation aligns with the SARSA (on-policy) algorithm than with standard Q-learning (off-policy). As training progresses, the Q-values become optimal under suitable conditions, enabling the agent to act optimally in the environment Fig. 2.

**Algorithm 2:**
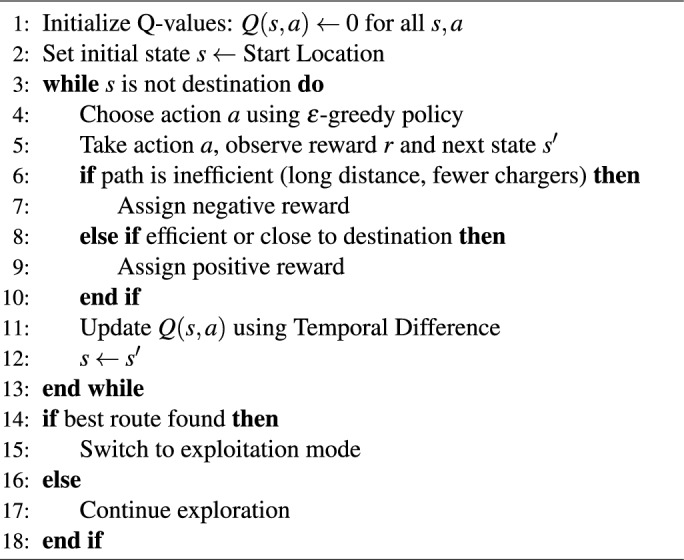
Reinforcement Learning-Based EV Routing Algorithm.

The Learning Algorithm of Double Q-Learning involves two agents. This is because an overestimation in values occurs in Q-Learning model when the same set of Q-values are used for selecting and evaluating actions. To overcome this, Double Q-Learning model uses two sets of Q-values, $Q_1(s, a)$ and $Q_2(s, a)$, and decouples the action selection from action evaluation during the learning process. The Temporal differences for 2 set of values is calculated in Equations (13) and (14). The agent chooses actions using an $\epsilon$-greedy policy based on the average of the two sets of Q-values and updates one set of values for selection and the other for evaluation as given in Equations (15) and (16).

13 $$\begin{aligned} & \delta _1 = r + \gamma Q_2\left( s', \arg \max _{a'} Q_1(s', a')\right) - Q_1(s, a) \end{aligned}$$

14 $$\begin{aligned} & \delta _2 = r + \gamma Q_1\left( s', \arg \max _{a'} Q_2(s', a')\right) - Q_2(s, a) \end{aligned}$$

15 $$\begin{aligned} & Q_1(s, a) \leftarrow Q_1(s, a) + \alpha \, \delta _1 \end{aligned}$$

16 $$\begin{aligned} & Q_2(s, a) \leftarrow Q_2(s, a) + \alpha \, \delta _2 \end{aligned}$$

**Fig. 3:**
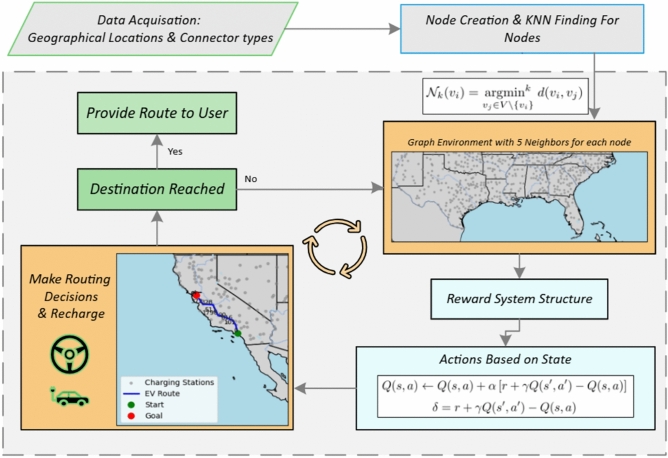
Overall Process Flow of EV Route Finding using RL Models. Maps generated using Cartopy v0.24.1^25^ with Natural Earth map data^26^.

Figure 3 illustrates the workflow of the RL-based routing models. The agent learns to find optimal routes on the graph structure by updating Q-values based on observed states and actions until the destination is reached. The graph is first constructed using a K-nearest neighbor (KNN) approach, after which the model learns routing policies through the defined reward system. To improve real-world feasibility, an additional distance-based optimization step is applied by selecting charging nodes within a range of 175–250 km. The initial route generated by the reinforcement learning agent represents node-to-node traversal across the charging station graph. Because stations are connected using a KNN structure, the learned route may include intermediate nodes with shorter distances. Therefore, a filtering stage is applied to skip unnecessary intermediate nodes and enforce realistic charging intervals (175–250 km). In rare cases, shorter segments may still occur when the network topology or charging station availability does not permit a feasible node within the preferred range. This range reflects practical EV operation, where drivers typically utilize only a portion of the nominal 300–400 km vehicle range to maintain a safety margin under real-world conditions.

### Implementations of improvised A* and Dijkstra models

#### Improvised A* Algorithm

The A* search algorithm is one of the traditional models used in routing systems to figure out efficient routes. Here, we use an evaluation function defined in Equation (17) to determine the most promising node to explore next.

17 $$\begin{aligned} f(n) = g(n) + h(n) \end{aligned}$$

where *f*(*n*) is the total estimated cost of the cheapest solution through node *n*, *g*(*n*) is the known cost from the start node to the current node *n*, *h*(*n*) is the heuristic estimate of the cost from node *n* to the goal node.

**Fig. 4:**
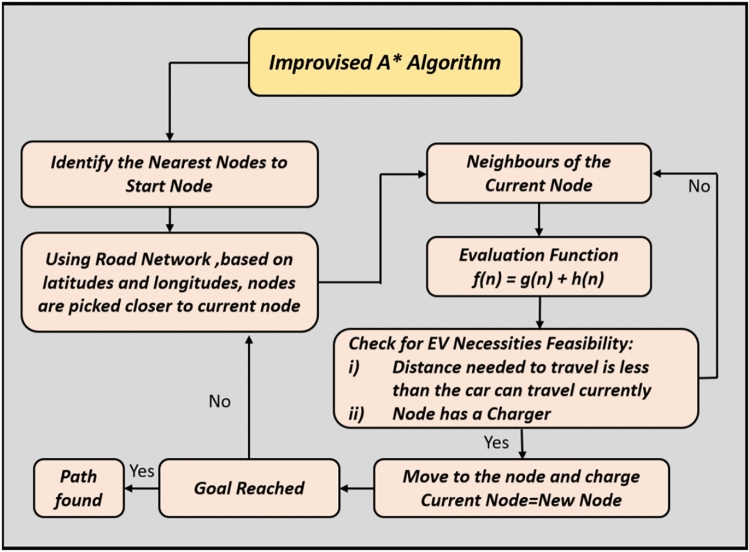
Improvised A* Algorithm Learning Process.

As shown in **Algorithm** 3, the Improvised A* algorithm adapts the standard A* framework for electric vehicle routing. While it retains the core principle of combining travel cost and heuristic estimates, it introduces EV-specific constraints such as limited battery range by preventing transitions to neighboring nodes if the required distance exceeds the available battery capacity. Additionally, as shown in Equation (18), charging is allowed only at nodes where charging stations are available. This ensures that the generated routes satisfy electric vehicle operational constraints and remain feasible for practical navigation Fig. 4.

18 $$\begin{aligned} & f(n, b) = g(n) + h(n) \quad \text {subject to } d(n, n') \le b \end{aligned}$$

19 $$\begin{aligned} & b' = {\left\{ \begin{array}{ll} B_{\text {max}}, & \text {if node } n' \text { has a charger} \\ b - d(n, n'), & \text {otherwise} \end{array}\right. } \end{aligned}$$

**Algorithm 3:**
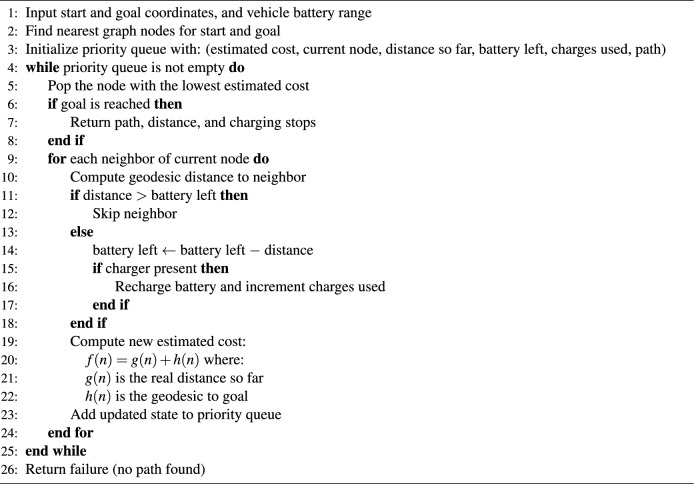
Improvised A* Algorithm for EV Routing

#### Improvised Djikstra algorithm

Dijkstra’s algorithm is employed to compute the path between two nodes in a graph, using edge weights that represent travel cost, typically distance. The algorithm explores paths in increasing order of cumulative cost from the source using Equation (20).

20 $$\begin{aligned} f(n) = g(n) \end{aligned}$$

The Improvised Dijkstra algorithm extends the standard Dijkstra framework by incorporating electric-vehicle constraints such as battery range and charging availability. Unlike the original version, it tracks the vehicle’s current battery level and the number of charging stops. Each state in the priority queue includes the current node, remaining battery, distance traveled, and charges used. When a node has a charging station, the battery is reset and the charge count increases. Edge costs use real geodesic distances between node coordinates. The update rule for computing the shortest distance between consecutive nodes is given in Equation (21).

21 $$\begin{aligned} d(v) = d(u) + \text {dist}(u, v), \quad \text {only if } \text {dist}(u, v) \le B_u \end{aligned}$$

## Results and discussion

This section provides a comprehensive analysis of the training dynamics and test-time performance of the proposed Primal–Dual Q model, evaluated against standard reinforcement learning baselines (Q-Learning and Double Q-Learning) as well as classical and energy-aware variants of A* and Dijkstra. The Testing was done with 10 different real-world routes across the USA and Canada using a graph-based charging station environment with realistic EV constraints such as connector types, port availability, and distance-based implications.

Each model is evaluated on four main metrics: *Total Distance Traveled*, *Number of Charging stations/ Nodes used throughout the route*, *Accuracy against the expected on-road distance*, *Estimated Energy Consumption*, and *Valid route*,i.e ,whether the model has adhered to the Electric vehicles constraints.

### Training performance of models for the first route

This section analyzes the training performance of proposed RL Models when finding optimal path for the first route.

#### Training analysis of primal-dual Q-learning

**Fig. 5:**
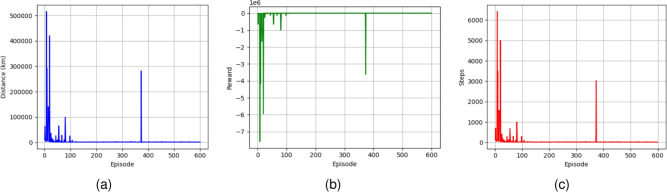
Primal Dual Q training metrics across episodes. (**a**) Distance travelled in each episode during Primal Dual Q training phase. (**b**) Accumulated reward in each episode during Primal Dual Q training phase. (**c**) Steps taken in each episode during Primal Dual Q training phase.

The Primal Dual Q model was trained for 600 episodes, and its training behavior shows a clear transition from high exploratory variability to stable exploitation. As seen in Fig. 5a(a) , the distance traveled per episode exhibits large fluctuations during the early exploratory phase, after which it rapidly stabilizes once the dual update mechanism begins enforcing consistency between primal and dual value estimates. The accumulated reward in Figure 5b(b) also shows high initial variance due to exploration across suboptimal routes but converges to consistently higher reward values as the model learns feasible and constraint-aware EV routes. Similarly, the number of steps taken per episode, shown in Figure 5c(c), decreases significantly after the early episodes, indicating that Primal Dual Q progressively identifies shorter, more efficient paths while respecting charging, connector, and distance constraints. Overall, the primal–dual update process enables faster stabilization and more reliable convergence compared to standard Q and Double Q methods.

#### Training analysis of Q-learning and double Q-learning

**Fig. 6:**
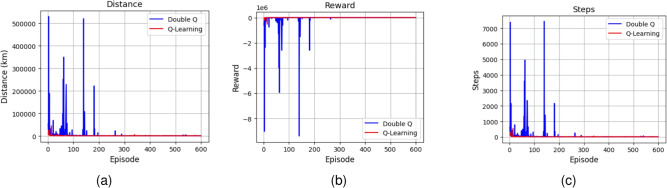
Comparison between Q-Learning and Double Q Learning training metrics across episodes. (**a**) Distance traveled (**b**) Accumulated reward (**c**) Steps taken.

Both Q-Learning and Double Q-Learning were trained for 600 episodes per route, and their learning behavior shows clear improvements across all metrics. As seen in Fig. 6a ,  b and c , the Q-Learning model begins with broad exploration but, after the exploration phase, consistently exploits shorter and more efficient routes. This is reflected in an increase in cumulative reward, a reduction in steps taken to reach the destination, and an improved selection of routes with favorable connector types and fewer penalties. The Double Q-Learning model exhibits similar trends. As shown in Figure 6, cumulative reward increases and the distance travelled decreases over training, indicating successful learning of efficient and constraint-aware routing. Its exploration period is slightly longer due to alternating updates across two Q-tables, enabling broader sampling before convergence. Overall, both models effectively adapt to EV constraints while improving route quality throughout training.

### Test performance and route visualization

This section analyzes the test performance and route visualization of proposed Primal-Dual Q-Learning Model alongside standard RL Models for the first route benchmarked against classical algorithms, A* and Dijkstra, which have also been used to find best paths for different routes with the improvised versions taking the Electric vehicle constraints into consideration and adhering to it. In the simulation environment, origin and destination locations are specified using geographic coordinates (latitude and longitude), which are mapped to the nearest nodes in the road network graph. This node-based representation allows the proposed routing framework to operate on any valid origin–destination pair within the network. The ten routes used in this study were selected as representative scenarios for evaluation and comparison with baseline algorithms, although the framework can accommodate arbitrarily generated routes.

#### Route evaluation: San Francisco to Portland

Each model was evaluated on a route between San Francisco and Portland (reference EV on-road distance: approximately 1020 km). Route analysis, shown in Table 3 show how each model approached the problem.

**Table 3:** Model Comparison: San Francisco to Portland.

Metric	Standard		Improvised		RL Models		Primal
	A*	Dijkstra	A*	Dijkstra	Q	Double Q	Dual Q
Distance (km)	1350.58	1444.56	1273.78	1273.78	1262.98	1375.88	1208.42
Accuracy (%)	75.55%	70.59%	79.73%	79.73%	80.76%	74.13%	84.41%
Charging Stops	13	15	13	13	12	10	11
Charge (kWh)	–	–	170	170	170	185	160
Valid Route	No	No	Yes	Yes	Yes	Yes	Yes

The Reinforement Learning models dynamically select charging stations with optimal port/connectors and making sure that distance traveled is least as possible, while Improvised A* and Dijkstra mostly favored the shortest geometric paths between nodes which have charging stations and neglected connector type preferences. It is infered that the proposed Primal Dual Q-Learning model has performed the best out of the considered models with 84.41% accuracy while Djikstra has performed with the least accuracy of 70.59%. A* and Dijkstra model routes are not valid in any cases, since they do not adhere to the electric vehicle constraints. The results of Improvised A* and Improvised Dijkstra are better than the standard models and have obtained same results in all cases since the conditions that they follow and helps monitoring the decision making with consideration of Electric Vehicle constraints are same.

#### Charging behavior

**Fig. 7:**
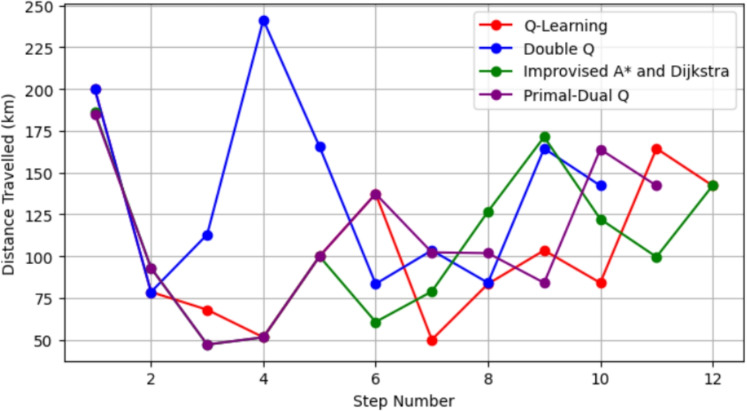
Distance traveled in each step taken across Valid models.

From Figure 7, it is clear that the Reinforcement Learning models make a little lesser stops and prefer nodes which are distanced quite far from each other, and only if they dont find one, they prefer closer nodes to avoid regular stopping intervals and charge only when necessary, improving real-world feasibility and lowering time consumption during charging.

#### Route visualization

**Fig. 8:**
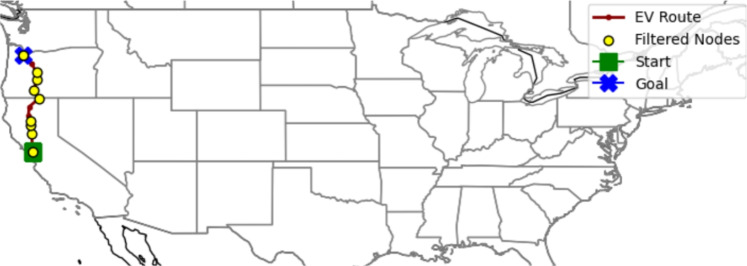
Representation of the route from San Franisco to Portland generated by Primal-Dual Q-Learning model on the USA map. Map generated using Cartopy v0.24.1^25^ with Natural Earth map data^26^.

**Fig. 9:**
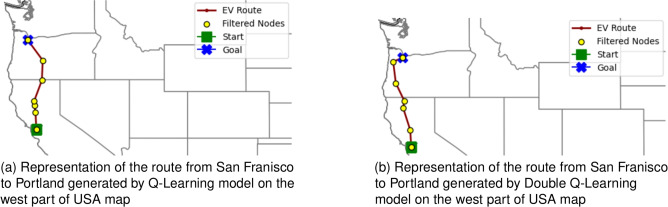
Routes generated by Q-Learning and Double-Q-Learning. Maps generated using Cartopy v0.24.1^25^ with Natural Earth map data^26^.

From Figure 8, it can be observed that the route proposed by the Primal–Dual Q-Learning model ensures better real-world feasibility, reduces unnecessary charging stops, and simplifies route planning. In comparison, Figs. 9a and b show that the routes generated by Q-Learning and Double Q-Learning require 12 and 10 nodes respectively to reach the destination.

As part of the RL route optimization process, the distance-based filtering step is applied, as illustrated in Fig. 8 and 9b , where the selected charging nodes are highlighted in yellow. These nodes are chosen such that the distance between consecutive charging points lies within the range of 175–250 km, which improves practical usability for EV drivers by reducing frequent stops and allowing charging only when necessary. The selected range reflects realistic EV operation, where drivers typically use only a portion of the nominal 300–400 km battery range to maintain a safety margin for real-world conditions such as traffic, terrain, and energy variability. In rare cases, shorter segments may still appear when the charging network topology does not provide a feasible station within the preferred range, requiring the agent to select the nearest available charging node.

**Fig. 10:**
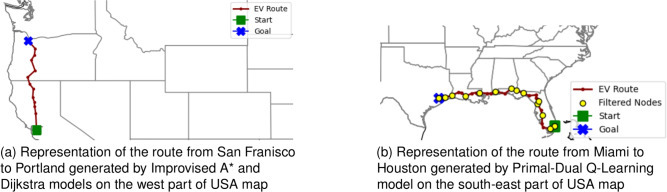
Comparative visualization of model-generated EV routes across two different origin–destination pairs. Maps generated using Cartopy v0.24.1^25^ with Natural Earth map data^26^.

This improves the practical usage of RL models in real life, improving real world feasibility and lowering time consumption during charging. Figure 10a shows the route proposed by the Improvised A* and Dijsktra which confirms that they look for closer and quicker solutions which results in little more number of charging stops but provide efficient results as well due to well structured heuristic function. Figure 10b shows a different route from Miami to Houston proposed by the Primal-Dual Q-Learning model which confirms that the model is efficient with longer routes as well.

### Quantitative comparison across proposed models

#### Individual route analysis across 10 routes

Table 5 gives the Complete Analysis of the routes proposed by each model. For an instance, in the route between Miami to Houston, the Primal-Dual Q-Learning model has 86.42% accuarcy (from Table 5), performing better than standard RL models and classical baselines, A* and Dijkstra and with optimization, the model got 93.30% accuracy on the route, outperforming Improvsied A* and Improvised Dijkstra as well. Dijkstra has performed the worse in this specfic route with 74.56% accuracy. It is infered from the individual route analysis that the Dijkstra model is worse than the A* Model and this to due the heuristic function being included in the A* model which helps in getting to the nodes faster than Dijkstra. As mentioned earlier, the Improvised A* and Improvised Dijkstra models give out same results as they follow conditions which have same considerations towards the Electric vehicle constraints. In many cases, the Improvised models do come out to be better due to the heuristic function containing range limit applied on them, which gets them to make different decisions than classical A* and Dijkstra models which do not make decisions based on range limits and electric vehicle constraints.

The proposed Primal-Dual Q-Learning model, on average, got a route which is better than A* and Dijkstra and Improvised versions of A* and Dijkstra models as noted in Table 5. The standard RL models also have also performed well and matched to the Improvised versions of A* and Dijkstra. They have managed to experience the environment around them with proper training better than the other models. This is crucially due to their reward system setup which makes them learn with proper conditions and getting maximum reward possible. The optimized results, shown in Table 5, reduce the number of charging stops while maintaining the overall route structure, achieving travel distances close to real road routes while adhering to EV constraints.

To evaluate the reliability of the proposed routing framework, each model was executed for 50 independent runs on the same origin–destination pair. For each run, the route produced by the reinforcement learning agent was converted into a real-world driving path using the OpenRouteService API. A run was considered successful if the resulting road distance was within $1.35\times$ the on-road distance between the start and destination. This threshold accounts for realistic deviations caused by road network constraints and charging station availability.

**Table 4:** Success rate comparison of routing models over 50 independent runs on the route from Los Angeles to San Francisco.

Model	Success Rate (%)
Q-Learning	70
Double Q-Learning	66
Primal–Dual Q-Learning	72
Primal–Dual Q-Learning + Optimization	**86**

Significant values are in [bold].

Table 4 presents the success rates obtained for the different routing approaches. The standard Q-learning model achieved a success rate of 70%, while Double Q-learning produced a slightly lower success rate of 66%. This behavior can be attributed to the deterministic structure of the routing environment and the reward shaping used in the model, which already limits overestimation bias and reduces the relative advantage of Double Q-learning. The proposed Primal–Dual Q-learning approach improves the success rate to 72%, demonstrating that incorporating a dual variable to balance reward and travel cost helps guide the policy toward more feasible routes. Furthermore, applying the distance-based route optimization step significantly improves performance. After enforcing practical charging intervals between 175–250 km, the success rate increases to 86%. While the same distance-based filtering procedure was also applied to the baseline methods to ensure a fair comparison, it resulted in only limited improvements in their overall routing performance. In contrast, the proposed Primal Dual Q-learning framework produces trajectories that are more amenable to post-processing, allowing the filtering stage to more effectively reduce redundant stops and, in several cases, total distance. This leads to routes that better align with practical EV charging behavior. Therefore, the optimized results are presented for the proposed method, where the impact of this step is most meaningful. This result indicates that the optimization stage effectively removes unnecessary intermediate nodes and produces routes that better align with realistic EV charging behavior.

**Table 5:** Comparison of Models Across Routes with Performance Metrics.

Route	On-Road	Metric	Standard		Improvised		RL Models		Proposed Primal-Dual Q	
	Distance		A*	Dijkstra	A*	Dijkstra	Q	Double Q	Primal-Dual	Optimized
	(km)								Q Adaptive	
San Francisco to Portland	1020	Distance (km)	1350.58	1444.56	1273.78	1273.78	1262.98	1375.88	1208.42	1203.04
		Accuracy (%)	75.55%	70.59%	79.73%	79.73%	80.76%	74.13%	84.41%	84.79%
		Charging Stops	13	15	13	13	12	10	11	8
		Charge (kWh)	–	–	170	170	170	185	160	160
		Valid Route	No	No	Yes	Yes	Yes	Yes	Yes	Yes
Los Angeles to San Francisco	610	Distance (km)	763.72	972.11	833.72	833.72	763.72	787.71	784.99	658.79
		Accuracy (%)	79.87%	62.75%	73.33%	73.33%	79.87%	77.41%	77.70%	92.59%
		Charging Stops	8	10	8	8	8	8	7	4
		Charge (kWh)	–	–	110	110	105	105	105	90
		Valid Route	No	No	Yes	Yes	Yes	Yes	Yes	Yes
Miami to Houston	1909	Distance (km)	2316.77	2563.62	2192.25	2192.25	2271.90	2221.42	2208.97	2046.18
		Accuracy (%)	82.40%	74.56%	87.08%	87.08%	84.03%	85.94%	86.42%	93.30%
		Charging Stops	22	29	23	23	23	21	21	11
		Charge (kWh)	–	–	295	295	305	300	300	275
		Valid Route	No	No	Yes	Yes	Yes	Yes	Yes	Yes
Orlando to Atlanta	710	Distance (km)	926.06	1110.53	925.16	925.16	930.12	925.07	930.57	916.36
		Accuracy (%)	76.67%	63.93%	76.74%	76.74%	76.33%	76.75%	76.30%	81.08%
		Charging Stops	12	13	11	11	11	11	11	7
		Charge (kWh)	–	–	125	125	125	125	125	125
		Valid Route	No	No	Yes	Yes	Yes	Yes	Yes	Yes
Dallas to Houston	390	Distance (km)	538.54	811.15	538.54	538.54	538.55	546.87	538.94	493.38
		Accuracy (%)	72.42%	48.08%	72.42%	72.42%	72.42%	71.32%	72.36%	79.05%
		Charging Stops	7	12	7	7	6	7	6	4
		Charge (kWh)	–	–	70	70	75	75	75	65
		Valid Route	No	No	Yes	Yes	Yes	Yes	Yes	Yes
Washington to Atlanta	1025	Distance (km)	1490.42	1441.12	1312.62	1312.62	1383.49	1384.97	1305.06	1136.01
		Accuracy (%)	69.77%	71.13%	78.09%	78.09%	74.09%	74.01%	78.54%	90.23%
		Charging Stops	16	20	14	14	14	14	13	6
		Charge (kWh)	–	–	175	175	185	185	175	155
		Valid Route	No	No	Yes	Yes	Yes	Yes	Yes	Yes
Las Vegas to Seattle	1810	Distance (km)	2499.70	3206.28	2425.67	2425.67	2502.62	2485.52	2395.82	1994.87
		Accuracy (%)	72.41%	56.45%	74.62%	74.62%	72.32%	72.82%	75.55%	90.73%
		Charging Stops	19	31	19	19	17	15	22	11
		Charge (kWh)	–	–	330	330	340	335	325	270
		Valid Route	No	No	Yes	Yes	Yes	Yes	Yes	Yes
Kansas to Oklahoma	565	Distance (km)	796.67	1110.90	750.63	750.63	750.62	778.47	727.49	616.27
		Accuracy (%)	70.92%	50.86%	75.27%	75.27%	75.27%	72.58%	77.66%	91.68%
		Charging Stops	11	18	10	10	10	10	7	4
		Charge (kWh)	–	–	100	100	100	105	100	85
		Valid Route	No	No	Yes	Yes	Yes	Yes	Yes	Yes
Montreal to Philadelphia	732	Distance (km)	1024.83	1410.33	959.79	959.79	930.28	959.79	960.90	802.08
		Accuracy (%)	71.43%	51.90%	76.27%	76.27%	78.69%	76.27%	76.18%	91.26%
		Charging Stops	11	14	8	8	7	8	7	4
		Charge (kWh)	–	–	130	130	125	130	130	110
		Valid Route	No	No	Yes	Yes	Yes	Yes	Yes	Yes
Dallas to Oklahoma	330	Distance (km)	463.53	569.07	404.98	404.98	405.33	404.98	404.97	337.45
		Accuracy (%)	71.19%	58.92%	81.49%	81.49%	81.41%	81.49%	81.49%	97.79%
		Charging Stops	6	7	5	5	4	5	4	3
		Charge (kWh)	–	–	55	55	55	55	55	45
		Valid Route	No	No	Yes	Yes	Yes	Yes	Yes	Yes

**Table 6:** Average Results Across 10 Routes.

Metric	A*	Dijkstra	Improvised A*	Improvised Dijkstra	Q-Learning	Double Q	Primal-Dual Q	Optimized Primal-Dual Q
Accuracy (%)	74.26%	60.92%	77.50%	77.50%	77.52%	76.27%	78.66%	89.25%
Valid Route	No	No	Yes	Yes	Yes	Yes	Yes	Yes

#### Performance summary for all routes

From Table 6, which reports the average accuracy across ten real-world routes, the Primal–Dual Q-Learning model consistently achieves the highest performance. It surpasses standard RL methods, exceeds A* by approximately 3–4%, and outperforms Dijkstra by more than 15%. Notably, it also delivers better performance than the improvised variants of A* and Dijkstra, producing routes that are both more efficient and better aligned with EV charging and constraint requirements.

The comparison in Table 6 highlights the clear advantages of the proposed Primal–Dual Q Learning framework over both classical and standard RL baselines. While Q-Learning and Double Q-Learning already outperform traditional A* and Dijkstra by learning the long-term effects of charger spacing, SoC limits, and station quality, they still rely on a fixed reward structure that cannot dynamically adjust to constraint violations. The Primal-Dual Q model addresses this limitation through its Lagrangian-driven adaptive weighting, allowing the agent to modulate the penalty on energy-constraint violations during training. This enables more stable convergence and more deliberate route selection, resulting in higher average accuracy (78.66%) than both standard RL and improvised graph-search methods. Moreover, the Optimized Primal–Dual Q variant shows the strongest overall performance at 89.25%, demonstrating that primal–dual adaptation combined with selective node optimization yields routes that are not only shorter but also more realistic and EV-feasible. Unlike classical methods, which make greedy distance-based decisions, and unlike standard RL agents, which balance reward components with static weights, the proposed primal-dual formulation actively re-weights cost and reward signals based on real-time constraint satisfaction, producing routes that better reflect real EV driving behavior across diverse long-distance scenarios.

**Table 7:** Ablation Study on the San Francisco to Portland Route.

Model	Distance (km)	Charging Stops	Accuracy (%)
Q-Learning (Baseline)	1262.98	12	80.76
Primal-Dual Q (Full Reward)	**1203.04**	**8**	**84.79**
Primal-Dual Q (Simplified Reward)	1474.17	–	69.19

Significant values are in [bold].

To evaluate the contribution of the reward formulation, an ablation experiment was conducted using a simplified reward function that only penalizes travel distance. As shown in Table 7, removing the additional reward components significantly degrades routing performance, resulting in longer routes and lower efficiency. The full reward formulation incorporates factors such as progress toward the destination, charging station compatibility, and penalties for revisiting nodes, which collectively guide the agent toward more feasible and efficient EV routes. These results demonstrate that reward shaping plays a crucial role in improving convergence stability and routing quality.

**Table 8:** Sensitivity analysis of DBSCAN epsilon parameter for charging station clustering on San Francisco–Portland route.

DBSCAN Epsilon (km)	Number of Charging Stops
10	21
20	11
30	8

To evaluate the impact of the DBSCAN clustering parameter on routing performance, a sensitivity analysis was conducted by varying the epsilon value used to group nearby charging stations. Table 8 presents the number of charging stops required for a representative route between San Francisco and Portland under different epsilon values. When the epsilon value is small (10 km), the clustering process produces many fragmented station groups, which increases the number of nodes in the routing graph and results in more frequent charging stops. Increasing the epsilon to 30 km significantly reduces the number of stops; however, this larger radius tends to over-aggregate nearby stations and reduces the spatial resolution of the charging network representation. An epsilon value of 20 km provides a balanced configuration, maintaining sufficient spatial detail in the charging station graph while still reducing redundancy in closely located stations. This setting preserves realistic routing options while avoiding excessive clustering or fragmentation, and was therefore selected for the experiments reported in this study.

## Implications for transportation systems and infrastructure planning

The results of this study highlight several important implications for the evolving EV transportation landscape. As EV adoption accelerates, reliable long-distance travel has become a defining factor in shaping user confidence, infrastructure requirements, and system-wide mobility patterns. The proposed primal–dual reinforcement learning framework demonstrates that data-driven routing strategies can meaningfully enhance trip reliability by identifying feasible and energy-aware paths across vast, geographically diverse networks. This improvement directly contributes to reducing range anxiety, enabling EV users to navigate intercity corridors with greater certainty and fewer unplanned detours, thereby strengthening the perceived practicality of EV travel.

Beyond individual trip optimization, the routing behavior observed in this study offers insights into infrastructure sufficiency across regions. Patterns in route selection and charging-stop distribution reflect how effectively current public charging networks support long-distance mobility. Routes that repeatedly divert from the shortest geometric path or encounter sparse charging options can help reveal spatial gaps in network coverage. These insights are valuable for transportation planners seeking to identify underserved corridors or prioritize areas for future charging deployment, especially as cross-border and interstate electric mobility becomes increasingly common.

The framework also has implications for charging-station operations. By generating routes that minimize redundant stops and avoid overly constrained areas of the network, the method can support more balanced utilization of charging facilities. Such load distribution can help mitigate congestion, reduce wait times, and alleviate peak demand pressures on the electrical grid. As charging infrastructure grows, intelligent routing systems that guide travelers toward efficient, predictable journeys will play an important role in maintaining network stability and ensuring equitable access.

Additionally, the lightweight and interpretable nature of the proposed approach makes it suitable for integration into navigation platforms, fleet-management systems, and public route-planning tools. Unlike methods that require deep neural architectures, the present framework relies on transparent value-based learning and real-world infrastructure data, enabling practical deployment at scale. Its performance across long-distance routes demonstrates the potential to enhance trip-planning services and to support transportation agencies in assessing corridor-level EV readiness. Collectively, these implications underscore the broader transportation-system relevance of the approach, extending beyond algorithmic performance to inform infrastructure planning, operational efficiency, and traveler experience.

## Comparative analysis

The differences observed between the proposed Primal–Dual approach and the standard Q-learning baseline help illustrate the role of the dual update within the learning process. In the baseline formulation, the agent relies solely on a reward-driven signal, which naturally promotes routes that are geometrically shorter or that produce higher immediate progress toward the destination. While effective in well-connected regions, this tendency often leads to infeasible choices when charging opportunities are sparse, since the agent does not explicitly account for the energy constraints that govern long-distance EV travel. This behavior is reflected in the baseline results, where Q-learning occasionally converges to paths that appear efficient but cannot be executed with the available state of charge Table. 9.

**Table 9:** Comparison of Existing EV Routing Approaches with the Proposed Primal–Dual Q-Learning Framework.

Methodology	Novelty Gap / Limitation	Advantage of Proposed Primal–Dual Q Approach
Cluster-based Route Planning^3^	Clusters nodes to improve travel time but does not explicitly model EV battery constraints or charging feasibility.	The proposed method learns energy-aware routing policies where state-of-charge (SoC) constraints directly influence route decisions.
FASTSim Energy Consumption Model^4^	Focuses on estimating EV energy consumption but does not integrate energy constraints into routing policy learning.	Energy feasibility is incorporated directly into the reinforcement learning environment, allowing the agent to jointly learn routing and charging decisions.
Topography-aware Energy Routing^5^	Accounts for terrain and battery degradation but relies on deterministic optimization rather than adaptive decision learning.	The proposed RL framework dynamically adapts routing strategies through interaction with the environment while maintaining energy feasibility constraints.
Charging Strategy Evaluation Study^6^	Primarily evaluates charging strategies and infrastructure usage rather than optimizing route selection under energy constraints.	The proposed framework performs end-to-end route optimization considering charger locations, SoC limits, and sequential decision-making.
Dynamic Programming for Power Delivery EVs^7^	Designed for EV-based power delivery to isolated regions rather than general transportation routing scenarios.	Our approach addresses real transportation networks and long-distance EV navigation using reinforcement learning.
Event-driven Charging Price Routing^8^	Optimizes routes based on electricity pricing events but does not explicitly enforce battery feasibility during routing.	The proposed model balances travel efficiency and energy feasibility through an adaptive primal–dual reward formulation.
Crowd-sensed EV Navigation^9^	Uses crowd-sensed traffic information but lacks integrated battery-aware routing optimization.	The proposed framework integrates SoC constraints, charging availability, and routing decisions within a unified learning environment.
Energy Consumption Law-based Planning^10^	Uses analytical energy consumption models but lacks adaptive learning for sequential routing decisions.	The RL agent learns routing strategies directly from interaction with the road network and charging infrastructure.
Ant Colony Optimization for EV Routing^11^	Metaheuristic approach relying on pheromone updates and static heuristics, which may not adapt well to large-scale networks.	Reinforcement learning continuously updates action values through environment interaction, improving adaptability to different routing conditions.
EV Routing with Charging Demand Modeling^12^	Focuses on vehicle routing problem formulations with charging constraints but relies on traditional optimization methods.	The proposed approach learns routing policies through reinforcement learning, enabling adaptive decision-making across diverse routes.
Genetic Algorithm-based Route Optimization^13^	Genetic algorithms require extensive parameter tuning and may struggle with scalability in large routing graphs.	Tabular Q-learning with a primal–dual update offers a lightweight and interpretable alternative with lower computational overhead.
Collaborative Charging Scheduling^14^	Emphasizes charging station scheduling and grid coordination rather than route-level learning.	The proposed method prioritizes route feasibility and efficiency by integrating charger availability directly into routing decisions.
Charging Slot Reservation Routing^15^	Relies on predefined reservation mechanisms and traffic conditions rather than adaptive policy learning.	Reinforcement learning enables flexible charger selection and route planning based on evolving state-of-charge conditions.
EV Health Monitoring and Range Prediction^16^	Focuses on battery health monitoring and range estimation rather than routing optimization.	The proposed framework incorporates range constraints directly into the route planning decision process.
Deep RL for EVRPTW^17^	Uses deep neural networks with high computational cost and training complexity.	The proposed tabular primal–dual Q-learning approach provides a lightweight and interpretable alternative while maintaining constraint awareness.
Spot-price and Multi-objective Routing^18^	Optimizes travel objectives based on electricity pricing but relies on predefined optimization rules.	Our approach introduces adaptive Lagrangian weighting through a primal–dual learning update that dynamically balances reward and constraint costs.
Deep RL with Two-stage Training^19^	Requires complex neural policy training and large computational resources.	The proposed framework avoids heavy neural architectures and achieves effective routing using interpretable tabular reinforcement learning.

SoC = State of Charge, RL = Reinforcement Learning.

In contrast, the Primal–Dual framework incorporates an adaptive penalty that becomes more influential whenever the agent encounters low-charge situations or transitions that threaten energy feasibility. The dual update increases the relative importance of the cost-based value function in these situations, guiding the agent toward actions that maintain viable state-of-charge levels while still pursuing efficient overall progress.

The performance gains observed in the route evaluations highlight the effect of this mechanism: the combined model avoids infeasible segments more reliably, requires fewer corrective detours, and identifies routes that strike a more appropriate balance between travel efficiency and charging sustainability. These outcomes indicate that the dual update is the primary factor enabling consistent learning across large, sparsely charged networks, and the Q-learning baseline implicitly serves as a meaningful ablation demonstrating the limitations of a reward-only strategy.

Compared to existing EV routing research, which primarily relies on static heuristics, predictive models, or computationally expensive metaheuristics, the proposed reinforcement-learning-based framework introduces a more adaptive and interpretable methodology for long-distance EV navigation. Classical graph-search algorithms such as A* and Dijkstra^3,12^ produce efficient geometric paths but cannot incorporate SoC limits, charger availability, or dynamic penalty adjustments. Metaheuristic approaches based on PSO, GA, or ant-colony optimization^10,11,13^ improve flexibility but require extensive parameter tuning, high computational cost, and lack real-time adaptability. Energy- and consumption-aware models in prior work—including cluster-based routing^3^, FASTSim-based energy estimation^4^, and topography-sensitive planning^5^—offer partial improvements but do not tightly couple the routing process with constraint feedback during execution. Similarly, studies focusing on charging strategies^6,14^, off-grid delivery^7^, dynamic pricing^8^, or crowd-sensed navigation^9^ often treat routing and energy management as separate tasks. This separation limits adaptability when encountering sparse infrastructure or varying charger characteristics. Approaches such as GRU-based forecasting or hybrid predictive-routing models^18^ provide useful demand estimation but do not perform policy learning. Deep reinforcement learning models for EVRPTW^17^ achieve strong performance but require significantly more computational overhead and are not optimized for large, discrete road-charging graphs.

In contrast, our framework integrates routing and energy-awareness into a unified reinforcement learning formulation. Standard Q-Learning and Double Q-Learning enable the agent to learn long-term consequences of charger spacing, SoC constraints, and detour penalties directly from interaction, without relying on hand-crafted heuristics or metaheuristic tuning. However, fixed reward weights limit their adaptability when constraint severity fluctuates across regions. The proposed Primal-Dual Q Learning model addresses this limitation by introducing a Lagrangian-driven adaptive weighting mechanism. Unlike prior energy-aware or predictive systems, the primal-dual formulation updates a dual variable during training to automatically adjust the importance of energy constraint violations. This enables more stable convergence when navigating sparse-charging segments and improves charging-aware decision making compared to standard RL agents. As demonstrated in the experimental results, the Primal-Dual Q model consistently outperforms classical algorithms, improvised variants, and baseline RL methods, offering higher route accuracy, fewer stops, and improved feasibility across diverse real-world routes.

## Conclusion and future scope

This work set out to examine the efficiency of reinforcement learning models against the benchmarked models, A* and Dijkstra in the field of EV route planning. And from the analysis, it is infered that the proposed Primal-Dual Q-Learning with adaptive weighting is indeed better at providing optimal paths with accuracy of 78.66% than classical A* and Dijkstra and the standard RL models and with optimization, it has outperformed the standard and improvised models by a massive margin, providing routes with accuracy of 89.25%, indicating their strong real world feasiblity with proper implementation. Thus, the proposed reinforcement learning framework provides a robust, flexible, and intelligent alternative for next-generation electric vehicle navigation systems. Overall, by combining lightweight tabular RL with a primal–dual adaptive mechanism, the proposed framework provides a scalable, interpretable, and infrastructure-aware EV routing solution that bridges the gap between static optimization methods and computationally heavy deep reinforcement learning approaches. It enables real-time, constraint-sensitive routing decisions suitable for modern EV navigation and intelligent transportation systems.

Future research can extend this framework by incorporating stochastic variables such as real-time traffic conditions and dynamic congestion, along with integrating terrain-aware energy consumption models and predictive estimates of charging-station occupancy. Incorporating driver-adaptive energy models may further improve SoC prediction accuracy. Hybrid strategies that combine the stability of classical graph-search methods with the adaptability of reinforcement learning—especially primal–dual or other constrained RL approaches—could enhance robustness in dynamic or infrastructure-limited settings. Future research can also explore advanced policy-gradient reinforcement learning methods, such as Proximal Policy Optimization (PPO), which may further enhance the scalability and adaptability of the routing framework in larger and more dynamic transportation networks. Expanding the framework to multi-vehicle coordination, grid-aware charging decisions, and large urban networks also offers promising directions for improving system efficiency and real-world deployment.

## Data Availability

The dataset used in this study is publicly available, and its source is cited in the references. The implementation code developed for this work is publicly available in a GitHub repository (https://github.com/Sarvesh-Ram-Kumar/Electric-Vehicle-Route-Optimization-on-Real-World-Charging-Networks) to support reproducibility of the results reported in this study.
